# Prediction is Production: The missing link between language production and comprehension

**DOI:** 10.1038/s41598-018-19499-4

**Published:** 2018-01-18

**Authors:** Clara D. Martin, Francesca M. Branzi, Moshe Bar

**Affiliations:** 1 BCBL. Basque Center on Cognition, Brain and Language, Paseo Mikeletegi 69, 20009 San Sebastian, Spain; 2 IKERBASQUE, Basque Foundation for Science, Maria Diaz de Haro 3, 48013 Bilbao, Spain; 3 Neuroscience and Aphasia Research Unit (NARU), Division of Neuroscience and Experimental Psychology, School of Biological Sciences, University of Manchester, Zochonis Building, Brunswick Street, Manchester, M13 9PL UK; 4 Bar-Ilan University, Gonda Brain Research Center, Building 901, Ramat-Gan, 5290002 Israel

## Abstract

Language comprehension often involves the generation of predictions. It has been hypothesized that such prediction-for-comprehension entails actual language production. Recent studies provided evidence that the production system is recruited during language comprehension, but the link between production and prediction during comprehension remains hypothetical. Here, we tested this hypothesis by comparing prediction during sentence comprehension (primary task) in participants having the production system either available or not (non-verbal versus verbal secondary task). In the primary task, sentences containing an expected or unexpected target noun-phrase were presented during electroencephalography recording. Prediction, measured as the magnitude of the N400 effect elicited by the article (expected versus unexpected), was hindered only when the production system was taxed during sentence context reading. The present study provides the first direct evidence that the availability of the speech production system is necessary for generating lexical prediction during sentence comprehension. Furthermore, these important results provide an explanation for the recruitment of language production during comprehension.

## Introduction

Recent studies have provided evidence for a potential role of production processes in language comprehension^1–4^, but what exactly is the link between production and comprehension is a central topic in language sciences and remains to be determined^5^. Based on several recent frameworks, this missing link could be prediction. Listeners constantly predict upcoming information during language comprehension (to facilitate comprehension and dialogue), and such predictions are accompanied by covert production^6–9^. Except some indirect support in the literature showing that language production skills (category fluency task, production vocabulary) and prediction are related^10–14^, there is no direct evidence so far that the production system is necessary for prediction during comprehension (see^15^ for review). Providing experimental evidence for this claim would generate important knowledge regarding the production-comprehension link and consequently improve our understanding of the neurocognitive foundations of human communication.

In the present study, we measured lexical prediction during sentence comprehension when taxing the production system. The rationale was that if production is mandatory for prediction, then prediction should vanish when the availability of the production system is reduced. This was done by preventing inner speech through articulatory suppression (AS; e.g., uttering a certain syllable repeatedly while completing the primary task^16^). If performance on the primary verbal task relies on inner speech, it should be significantly impaired by AS because articulation of irrelevant information prevents subvocal rehearsal of the verbal input^17^.

Lexical prediction can be measured through event-related potential (ERP) responses derived from electrophysiological recording during sentence reading. Importantly for our goals, there is consistent evidence that the mean amplitude of the N400 negative ERP component is sensitive to lexical predictability: The less predictable a word is, the more negative the ERP N400 component. For instance, reading “The king wore on his head…” leads to lexical prediction of the noun-phrase “a crown”. The sentence ending “a hat” elicits a larger N400 component on the noun (reflecting difficulty in unexpected target noun integration), and, importantly, a larger N400 component on the article (when expected and unexpected nouns are of different gender and thus preceded by differently gender-marked articles; “una corona”/a crown – “un sombrero”/a hat). Such ERP modulation (i.e., larger amplitude for ERP component elicited by the unexpected relative to expected article) has been repeatedly observed and interpreted as a marker of lexical prediction, by taking advantage of gender-marked determiners in Spanish^18–20^, gender-inflected adjectives in Dutch^21^ and phonological properties of English (indefinite article “a” changed to “an” if the following noun begins with a vowel^22,23^ but see^24^ for a lack of replication).

We compared three groups of participants reading highly constrained Spanish sentences containing expected *versus* unexpected noun-phrases (primary task). Lexical prediction effects were measured through ERP N400 modulations on the article (whose gender was congruent or not with that of the most expected target noun) and compared across the three groups differing in the secondary task. To test whether taxing the production system would reduce lexical prediction, the SP (Syllable Production) group was assigned a verbal secondary task (i.e., AS) preventing participants from using their inner speech (pronouncing the syllable/ta/once on every word display). As a control for double-tasking, the TT (Tongue-tapping) group was assigned a non-verbal secondary task similar to AS but without requiring verbalization (tapping the tongue loudly once on every word). As a control for auditory feedback perception (inherently happening in the SP group), the SL group was assigned a ‘Syllable Listening’ secondary task (listening to own voice pronouncing/ta/on every word). If the production system is necessary to build up predictions, the N400 expectation effect elicited by the article should be reduced in the SP group relative to the control groups. As a control for proper sentence processing and lexical integration, we expected a significant N400 effect on critical nouns in the three groups.

## Material and Methods

### Participants

Sixty Spanish native speakers took part in the experiment. They were randomly assigned to three groups. The sample size was chosen based on previous ERP studies reporting N400 effects in sentence processing^19,20,23^. Twenty participants (9 females; age range 19–30, mean: 25 ± 3) were assigned to the ‘Syllable Production’ (SP) group. Twenty participants were assigned to the ‘Tongue-tapping’ (TT) group. Two participants were removed from analyses because of large number of artefacts in electroencephalogram recording (more than 50% trials removed after artefact rejection). The final TT group consisted of 18 participants (11 females; age range 19–30, mean: 24 ± 3). Twenty participants were assigned to the ‘Syllable Listening’ (SL) group. For similar reasons than in the TT group, 2 participants had to be removed from analyses, the final TT group thus consisting of 18 participants (11 females; age range 19–30, mean: 23 ± 3). The three groups were matched on age (F[2,53] = 1.48, p = 0.24). All participants were right handed, their vision was normal or corrected to normal and they did not report any reading or neurological disorder. Participants all signed an informed consent form before taking part to the study that was approved by the BCBL ethics committee. The experiment was performed in accordance with relevant guidelines and regulations. They received a payment of 10€ per hour for their participation.

### Materials

Stimuli consisted of 100 sentence contexts with two possible critical noun-phrases (article + noun): expected or unexpected (e.g., “*El rey llevaba en la cabeza* *una corona/un sombrero* *antigua/antiguo” – “The king wore on his head an old crown*_*[Fem]*_*/hat*_*[Masc]*_”; see Table 1 for other examples of sentences). In 50 sentence contexts, the expected noun was masculine (“un/el + noun” expected noun-phrase) and the unexpected noun was feminine (“una/la + noun” unexpected noun-phrase). In the other 50 sentence contexts, the expected noun was feminine and the unexpected noun was masculine (all critical nouns were inanimate). The 200 sentences were divided into two lists of 100 and each participant was presented with one list (matched across groups). Sentence contexts and critical noun-phrases were used only once per list. Each list contained 50 expected and 50 unexpected noun-phrases. There were no semantic or syntactic violations as critical noun-phrases were always semantically and syntactically correct, albeit that one was more expected than the other (see Table 1). There were no gender violations such as in “la sombrero – the_[Fem]_ hat_[Masc]_” or “el corona – the_[Masc]_ crown_[Fem]_”. The target noun-phrase was never in sentence final position. Across sentences, the critical article was in position 13.1 (SD 3.7; range: 6–24) and followed by 2.2 (SD 1.1) extra words (range: 1–6).

**Table 1:** Examples of sentences. Critical expected/unexpected noun-phrases are depicted in red.

Antes de entrar al piso tuvo que quedar con el propietario para firmar el contrato/la escritura ante notario.
*Before entering the flat, I had to plan to meet the owner to sign the contract/the deed with a public notary*.
Cuando estés desorientado mira la brújula, porque siempre muestra el norte/la dirección y el camino.
*When you get disoriented, look at the compass which always shows the north/the direction and the path*.
Nunca sé dónde llevar mi móvil y mi cartera, tengo que comprarme un bolso/una mochila que combine con todo.
*I never know how to carry my mobile and my purse, I need to buy a bag/a backpack to carry it all*.
Para pedirle matrimonio se arrodilló ante ella y le dio un anillo/una joya brillante.
*To ask her to marry him, he knelt in front of her and gave her a sparkling ring/gem*.
Por precaución, siempre que cojas la moto debes ponerte el casco/la chaqueta integral/de piel.
*To be cautious, you should put on the integral helmet/the leather jacket each time you drive your motorbike*.
Cuando era joven tocaba la batería en un grupo/una banda famoso/famosa.
*When he was young, he used to play in a famous group/band*.
Desde la terraza del apartamento de la playa se podía ver el mar/la catedral y los surfistas/y el mar.
*From the terrace of the beach apartment once could see the see/the cathedral and the surfers/and the see*.
Para cortar la carne se necesita un cuchillo/una tabla de metal/y un cuchillo.
*To cut the meat once need a knife/a board of metal/and a knife*.
El rey llevaba en la cabeza una corona/un sombrero antigua.
*The king wore an ancient crown/hat on his head*.
El símbolo del catolicismo es la cruz/el pez en muchas iglesias.
*The symbol of Catholicism is the cross/the fish in many churches*.
La ropa está sucia, ponla en la lavadora/el suelo por favor.
*The clothes are dirty, put them in the washer/on the floor please*.
Los niños hacen castillos de arena en la playa/el patio durante el verano/el recreo.
*Kids build sand castles on the beach/in the playground during summer/recess*.
Acabo de salir de casa y no recuerdo si he cerrado la puerta/el armario cuando me he ido.
*I just left home and I cannot remember if I closed the door/the cupboard when I left*.
Cada invierno se hace una campaña para vacunar a la gente mayor contra la gripe/el virus común/de la gripe.
*Every winter a vaccination campaign against the common flu/the flu virus is organized for older people*.
Se despertó sudando y temblando, había tenido una pesadilla/un sueño terrible.
*He woke up sweating and shivering, he had a terrible nightmare/dream*.
Todo quedó a oscuras porque se había ido la luz/el sol de repente.
*Everything went dark because of a sudden lack of* (*the*) *light/sun*.

English translations are provided, below each sentence, in italic.

The mean cloze probability of expected and unexpected critical nouns was assessed by native speakers of Spanish (N = 20) who did not take part to the experiment. These participants were presented with sentences truncated before the critical noun-phrase and asked to complete the sentence with the first continuation that came to their mind. The cloze probability of a noun was defined as the percentage of times it was used as continuation. The mean cloze probability for expected nouns and for expected whole NPs was respectively 0.86 (SD 0.09; range 0.6–1), and 0.84 (SD 0.10; range 0.4–1); the mean cloze probability for unexpected words and unexpected NPs was 0.00 (SD 0.01; range 0.0–0.05), and 0.00 (SD 0.01; range 0.0–0.05). Expected nouns (and NPs) had larger cloze probabilities than unexpected nouns (and NPs; all ps < 0.001).

Within each list, expected and unexpected target nouns were matched (based on EsPal database^25^) for grammatical gender, word frequency, number of letters, number of neighbors, number of syllables, familiarity, imageability, concreteness, averaged position of the critical article in the sentence, and averaged number of words following the critical NP (see Table 2). Expected and unexpected target nouns only differed in cloze probability. Critical words were also balanced across lists on all the critical variables (Table 2).

**Table 2:** List of variables matched within and across lists.

List 1	Expect	Unexpect	p1	List 2	Expect	Unexpect	p2	pE	pU
Freq	1.55(*0.53*)	1.49(*0.60*)	0.63	Freq	1.57(*0.54*)	1.43(*0.61*)	0.24	0.84	0.62
N let	5.82(*1.81*)	5.90(*1.83*)	0.83	N let	6.08(*2.00*)	6.14(*2.16*)	0.89	0.50	0.55
N neig	6.58(*7.72*)	7.84(*8.05*)	0.43	N neig	6.96(*7.37*)	7.52(*8.07*)	0.72	0.80	0.84
N syll	2.42(*0.84*)	2.44(*0.79*)	0.90	N syll	2.48(*0.86*)	2.56(*0.93*)	0.66	0.72	0.49
Fam	6.10(*0.64*)	6.01(*0.49*)	0.46	Fam	6.02(*0.63*)	5.89(*0.70*)	0.34	0.55	0.34
Imag	5.67(*1.03*)	5.54(*0.91*)	0.53	Imag	5.80(*0.71*)	5.47(*1.11*)	0.08	0.46	0.73
Concr	5.48(*1.00*)	5.19(*1.00*)	0.17	Concr	5.34(*1.02*)	5.04(*0.93*)	0.15	0.49	0.46
CP N	0.85(*0.09*)	0.00(*0.01*)	0.00	CP N	0.87(*0.09*)	0.01(*0.02*)	0.00	0.23	0.14
CP NP	0.83(*0.11*)	0.00(*0.01*)	0.00	CP NP	0.85(*0.09*)	0.01(*0.02*)	0.00	0.35	0.14
Pos art	13.1(*3.7*)	13.0(*3.8*)	0.91	Pos art	13.0(*3.8*)	13.1(*3.7*)	0.91	0.91	0.91
N f-w	2.2(*1.1*)	2.3(*1.1*)	0.65	N f-w	2.3(*1.1*)	2.2(*1.1*)	0.65	0.65	0.65

**Expect** = Expected condition; **Unexpect** = Unexpected condition; **p1** = p values for t-tests comparing variables in expected *versus* unexpected conditions for List 1; **p2** = p values for List 2; **pE** = p values for t-tests comparing expected conditions in List 1 and 2; **pU** = p values for unexpected conditions.

**Freq** = mean Log-frequency; **N let** = number of letters; **N neig** = number of neighbors; **N syll** = number of syllables; **Fam** = familiarity; **Imag** = imageability; **Concr** = concreteness; **CP N** = cloze probability of the target noun**; CP NP** = cloze probability of the target noun-phrase; **Pos art** = averaged position of the critical article in the sentence; **N f-w** = average number of words following the target noun-phrase in the sentence.

### Experimental design

The EEG experiment was run in a soundproof electrically shielded chamber. Participants were seated in a chair, about sixty centimeters in front of a computer screen. Stimuli were delivered with the Presentation software (https://www.neurobs.com/). Participants had to read sentences displayed one word at a time (200 ms + 500 ms inter-stimulus blank interval) in the center of the computer screen, on a grey background. Sentence words were displayed in red until 3 words before the critical article and in white from 2 words before the critical article until the final word of the sentence. Each sentence was preceded by a fixation cross displayed for 2000 ms. The common instruction for the three groups was to read each sentence silently and to answer ‘yes’ or ‘no’ to the following comprehension question by pressing a YES or NO button on a keyboard. Comprehension questions were inserted after each sentence to keep participants engaged in the silent reading task, and to get a complete assessment of sentence comprehension (to make sure that some dual-tasks were not more disturbing than others in terms of sentence comprehension).

Apart from reading sentences for comprehension, participants received other instructions varying depending on the group they were assigned to. Participants in the SP group were asked to produce the syllable/ta/each time a red word was displayed, and to stop doing so when the words started to turn into white (2 words before the critical article). This way, we made sure that double-tasking was performed during reading the first words of the sentence (i.e., sentence context used to build up predictions) and that it stopped on the word preceding the target article. This was crucial to avoid contamination by muscular activity of the ERPs time-locked on the target article. Participants in the TT group were asked to perform tongue-tapping each time a red word was displayed, and to stop doing so on words displayed in white. Note that since sentences were presented one word at a time on the computer screen, regularity for the secondary task was provided by the regularity of word display with no need of including beats (as usually done in AS experiments; see^16^). Note also that the SP and TT groups performed a secondary task with similar cognitive burden, both including motor action and feedback perception. The only difference between the two tasks was in the “linguistic status” of the articulation and feedback, being a syllable in the SP group and a noise in the TT group. Finally, participants in the SL group were informed that, during reading, they were going to listen to their own voice pronouncing/ta/on each word displayed in red, and not anymore once the words turn into white. In order to do so, after signing consent form and before preparing the electrode cap, participants assigned to the SL group were asked to pronounce the syllable/ta/several times in front of a microphone. Ten different utterances of the syllable were then extracted and inserted to the program, so that each participant would listen to her own voice. Along the experiment, each word displayed in red was presented together with one of the 10 utterances, randomly assigned. Each utterance was displayed 360 ± 75 ms after the word onset (range 285–435 ms), randomly, in order to mimic latencies of feedback perception during/ta/production (SP group).

All participants were explicitly encouraged to focus on sentence comprehension and to try to avoid distraction from the second task. Participants were informed that in case they would have stopped performing the secondary task (in the case of SP and TT groups), the experimenter would have reminded them to continue. Note that no participant had to be reminded of the secondary task, probably because the red display of the first words of each sentence was a clear signal reminding the participants they had to start again the secondary task.

Stimuli were presented in four blocks of 25 sentences, with a small break between the blocks. A brief practice session included three sentences, and the corresponding yes-no questions. Overall, the experiment lasted one hour and 30 minutes on average.

### Electrophysiological recording and statistical analyses

Electrophysiological data were recorded from 27 TiN electrodes placed according to the 10–20 convention (Easycap; Fp1/2, F7/8, F3/4, FC5/6, FC1/2, T7/8, C3/4, CP1/2, CP5/6, P3/4, P7/8, O1/2, F/C/Pz). Additional electrodes were placed over the left (on-line reference) and right mastoids. A forehead electrode served as the ground. Four electrodes were placed around the eyes (VEOL, VEOR, HEOL, HEOR) in order to detect blinks and eye movements. Data were amplified (Brain Amp DC) with a bandwidth of 0.01–100 Hz, at a sampling rate of 250 Hz. Impedances were kept below 5 kOhm for the scalp electrodes and 10 kOhm for the eye electrodes. Recordings were off-line re-referenced to the average activity of the two mastoids and re-filtered with a 30 Hz low pass filter (48 dB/oct) and a 0.1 Hz high pass filter (12 dB/oct). Eye blink artifacts were corrected using the Gratton *et al*.’s procedure^26^, implemented in Brain Vision Analyzer 2.0 (Brain Products, München, Germany), and any remaining artifacts exceeding +/−100 μV were dismissed. On average 7.41% of epochs were considered artifacts. The number of dismissed epochs was slightly larger for unexpected relative to expected nouns (F[1,53] = 4.26, p = 0.044) with no difference across groups (Group effect: F[2,53] = 0.41, p > 0.250; Group × Expectation interaction: F[2,53] = 0.42, p > 0.250). In the SP group, the final number of trials was 46.2 on average (SD 3.9; range 38–50) in the Expected condition and 45.8 on average (SD 3.9; range 38–50) in the Unexpected condition. In the TT group, the final number of trials was 46.5 on average (SD 3.3; range 39–50) in the Expected condition and 45.5 on average (SD 4.3; range 36–50) in the Unexpected condition. In the SL group, the final number of trials was 47.1 on average (SD 3.2; range 39–50) in the Expected condition and 46.7 on average (SD 3.8; range 38–50) in the Unexpected condition. Epochs ranged from −200 to 1400 ms after the onset of the critical article (including critical article and noun display). Baseline correction was performed in reference to pre-stimulus activity (−200–0 ms) and epochs were averaged independently for each condition and participant. ERP components were defined based on the grand averages and analyzed in the time-window classically used to explore the N400 component in similar paradigms: 300–500 ms for both the N400 component following the presentation of the article and the presentation of the noun^19,23^. Analyses of the 2 N400 peaks were conducted in 9 regions: anterior-left (F3, F7, FC1 electrodes), anterior-medial (FP1, FP2, Fz), anterior-right (F4, F8, FC2), central-left (T7, FC5, CP5), central-medial (C3, Cz, C4), central-right (T8, FC6, CP6), posterior-left (P7, CP1, O1), posterior-medial (P3, Pz, P4) and posterior-right (P8, CP2, O2). Mean amplitudes were subjected to a repeated measures analysis of variance (ANOVA; Greenhouse-Geisser corrected) for each component with Expectation (expected; unexpected), Longitude (anterior; central; posterior) and Laterality (left; medial; right) as within-subject factors and Group (SP; TT; SL) as between-subject factor. Post-hoc analyses mainly focused on the expectation effect employing Bonferroni corrected t-tests.

### Data availability

The datasets generated during and/or analyzed during the current study are available from the corresponding author on reasonable request.

## Results

### Comprehension questions

Participants’ responses to the comprehension questions during the EEG session were high (SP group: 80 ± 0.07% accuracy, range 70–90%; TT group: 81 ± 0.08%, range 60–96%; SL group: 83 ± 0.08%, range 65–93%), revealing proper general comprehension of sentences, and importantly did not significantly differ in the three groups (F[2,53] = 0.93, p = 0.40, *ƞ*^2^ = 0.034).

Since response times in cognitive tasks are known to increase with larger cognitive burden^27,28^, we also compared reaction times across the three groups to obtain an indirect estimation of the cognitive load induced by the different secondary tasks. Mean reaction times to the comprehension questions did not significantly differ in the three groups (SP group: 3528 ± 855 ms; TT group: 3454 ± 625 ms; SL group: 3755 ± 1100 ms; F[2,53] = 0.57, p = 0.57, *ƞ*^2^ = 0.021).

Higher cognitive load has been associated to slower performance but also increased distraction and so increased variability in response^29,30^. Consequently, we can safely assume that if any of the secondary tasks was associated with higher cognitive load, performance would be more variable in the group undergoing this task (i.e., larger standard deviations in performance should be observed). Thus, we also explored variability in performance by computing the standard deviation in accuracy and reaction time for each participant, and testing whether those standard deviations were affected by the secondary task (i.e., differed across groups). Neither standard deviations in accuracy (SP group: 0.39 ± 0.05; TT group: 0.38 ± 0.07; SL group: 0.36 ± 0.06) nor standard deviations in reaction times (SP group: 1551 ± 779 ms; TT group: 1317 ± 401 ms; SL group: 1662 ± 886 ms) significantly differed across the three groups (F[2,53] = 1.11, p = 0.34, *ƞ*^2^ = 0.040 and F[2,53] = 1.07, p = 0.35, *ƞ*^2^ = 0.039 respectively).

### ERP data

**Figure 1:**
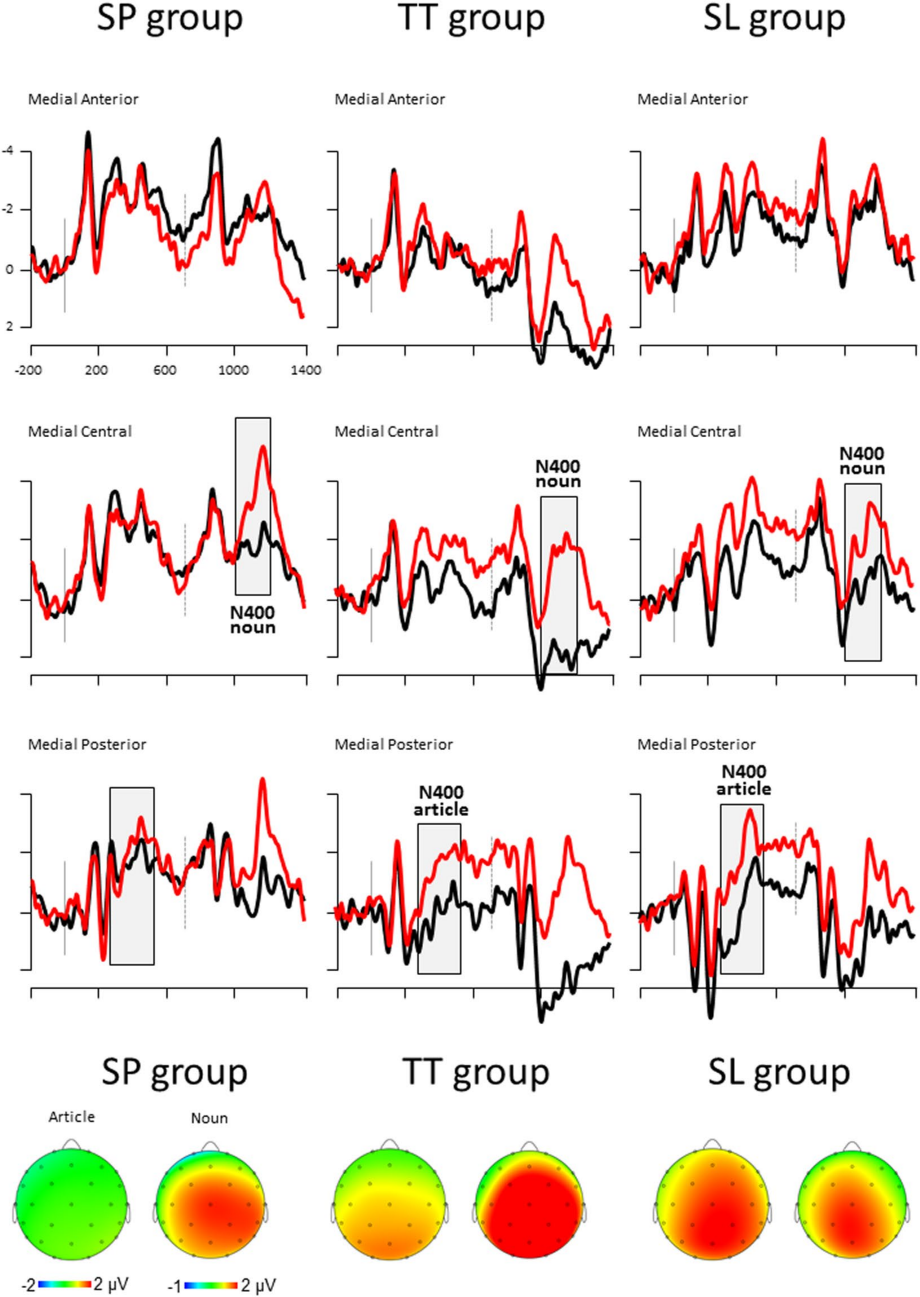
**Top panel:** Event-related potential results for the critical noun phrase, in the SP group (left panel), TT group (medial panel) and SL group (right panel). Time zero (vertical grey line) indicates the presentation of the critical article and time 700 ms (vertical grey dotted line) indicates the presentation of the critical noun. Black lines depict ERPs measured for expected noun-phrases; red lines depict ERPs measured for unexpected noun-phrases. ERPs measured over the Medial Anterior (FP1, FP2, Fz), Medial Central (C3, C4, Cz) and Medial Posterior (P3, P4, Pz) scalp. Grey areas indicate the time-windows used to measure the N400 wave elicited by the article (300–500 ms after the article onset) and the N400 wave elicited by the noun (300–500 ms after the noun onset). Negativity is plotted up. **Bottom panel:** Topographical maps of the N400 effect (expected minus unexpected conditions) elicited by the article and noun in the SP, TT and SL groups. Each map depicts the mean amplitude of the expected-unexpected difference in the 300–500 ms time-window following the critical word, from −2 to 2 μV for the article and from −1 to 2 μV for the noun (except for the noun in the TT group: from 0 to 3 μV).

The ERP pattern elicited by the critical noun-phrases is depicted in Fig. 1. The distribution of the late ERP component elicited by the article is consistent with the long-lasting effect that has been previously reported in similar experiments on lexical prediction, and consistently labeled N400 [e.g.^20,22,23^]. Whether such component should be assimilated to the classical N400 component or not is open to debate. Nevertheless, since the interpretation of our results does not depend on the component per se (but on the modulation of ERPs by expectation), we will use the N400 label in order to follow the literature.

#### N400 elicited by the article

Analyses revealed significant effects of Expectation (F[2,53] = 18.33, p < 0.001, *ƞ*^2^ = 0.257), Longitude (F[2,106] = 30.42, p < 0.001, *ƞ*^2^ = 0.365) and Laterality (F[2,106] = 22.14, p < 0.001, *ƞ*^2^ = 0.295) and no significant Group effect (F[2,53] = 1.98, p = 0.148, *ƞ*^2^ = 0.070). Importantly, the Expectation × Group interaction was significant (F[2,53] = 5.38, p = 0.007, *ƞ*^2^ = 0.169). Group × Longitude and Group × Laterality interactions did not reach significance (F[4,106] = 1.17, p = 0.328, *ƞ*^2^ = 0.042 and F[4,106] = 1.15, p = 0.335, *ƞ*^2^ = 0.042 respectively). Expectation × Longitude interaction was significant (F[2,106] = 7.32, p = 0.001, *ƞ*^2^ = 0.121) as well as Longitude × Laterality interaction (F[4,212] = 17.75, p < 0.001, *ƞ*^2^ = 0.251) and Expectation × Longitude × Laterality (F[4,212] = 4.33, p = 0.002, *ƞ*^2^ = 0.076). Other interactions did not reach significance (all ps > 0.05).

Post-hoc analyses of the Expectation × Group interaction revealed a significant expectation effect in the TT and SL groups (p = 0.037 and 0.002 respectively) and no such significant effect in the SP group (p > 0.99; see Fig. 1).

#### N400 elicited by the noun

Analyses revealed significant effects of Expectation (F[1,53] = 9.48, p = 0.003, *ƞ*^2^ = 0.152), Longitude (F[2,106] = 9.85, p < 0.001, *ƞ*^2^ = 0.157) and Group (F[2,53] = 3.64, p = 0.033, *ƞ*^2^ = 0.121). The Expectation × Group interaction was not significant (F[2,53] = 1.05, p = 0.358, *ƞ*^2^ = 0.038). Group × Laterality and Group × Laterality × Expectation interactions reached significance (F[4,106] = 3.64, p = 0.008, *ƞ*^2^ = 0.121 and F[4,106] = 2.83, p = 0.028, *ƞ*^2^ = 0.096 respectively). Group × Longitude × Laterality interaction was also significant (F[8,212] = 2.65, p = 0.009, *ƞ*^2^ = 0.091) as well as Expectation × Longitude × Laterality interaction (F[4,212] = 4.02, p = 0.004, *ƞ*^2^ = 0.070). Other main effects and interactions did not reach significance (all ps > 0.07).

Post-hoc analyses of the Group × Laterality × Expectation interaction revealed a significant expectation effect on the left, medial and right sites in the TT group (all ps < 0.001). The expectation effects was significant on the medial and right sites in the SP group (both ps < 0.001) but was not significant on the left sites (p = 0.086). The expectation effect was significant on the left sites in the SL group (p = 0.003) but was not significant on the medial and right sites (p > 0.99).

To summarize, the expectation effect on the critical article was significant in the two control groups (TT and SL groups) but did not reach significance in the SP group. The magnitude of this effect did not significantly differ between the TT and SL groups. The expectation effect on the critical noun was significant in the three groups (left lateralized in the SL group). The magnitude of this effect did not significantly vary across the three groups.

## Discussion

The aim of the present study was to determine whether the link between the production system and language comprehension is prediction^5^. To do so, we capitalized on recent frameworks arguing that prediction during comprehension is based on actual production. In other words, we hypothesized that the production-comprehension link might be explained, at least partly, by the mandatory role of production in prediction. To test this hypothesis, we explored whether the availability of the production system was indeed necessary for prediction during sentence comprehension. We measured the magnitude of the lexical expectation effect during sentence reading (N400 effect elicited by expected relative to unexpected noun-phrases) in three groups of participants differing in a simultaneous secondary task: syllable production (aimed to tax the production system by preventing subvocal rehearsal of the verbal input; SP group), tongue-tapping (aimed to mimic syllable production without taxing the production system; TT group) and syllable listening (aimed to mimic feedback perception inherently associated to syllable production in the SP group; SL group). We hypothesized that the expectation effect should be larger in the TT group relative to the SP group, if prediction requires availability of the speech production system. Plus, we hypothesized a larger expectation effect in the SL group relative to the SP group if taxing the production system – and not own voice feedback perception – was responsible for the reduced prediction in the SP group.

The results revealed that the expectation effect was reduced in the SP group relative to both the TT and SL groups. Participants in the TT and SL groups only actively predicted upcoming words during sentence reading (significant expectation effect on the critical article^18–20^). These findings show that taxing the production system (here, preventing subvocal rehearsal of the verbal input during sentence context reading) hinders prediction during sentence comprehension. Crucially, performing articulatory movements and perceiving associated feedback (TT group) or listening to own speech during reading (SL group) are not the factors responsible for the reduced expectation effect in the SP group. With the present experimental series we provide the first direct evidence for a strong and relevant implication of the production system in lexical prediction during sentence reading. It remains to be explored whether the production system plays a crucial role in other types of prediction (e.g., semantic, phonological prediction) and whether its major impact on prediction generalizes to other experimental settings. In fact, the production system might play a critical (even mandatory) role in ‘prediction by *simulation*’ (prediction based on own-body experience) and not in ‘prediction by *association*’ (prediction based on previous perceptual experience; see^9,15^). For now, our results support the view that the production system plays a critical role in lexical prediction during sentence comprehension^6,7,31,32^. Importantly, our results are not only relevant for models on language comprehension, but also for the main open and crucial question on the link between production and comprehension in language^5^. Going one step beyond previous studies showing that the production system is engaged during speech perception^1–4,33^, we show that such involvement of the production system during comprehension can be explained, at least partly, by its major role in prediction.

It could be argued that the reduction of prediction effects in the SP group was the consequence of larger cognitive burden in the syllable production secondary task, relative to the tongue tapping and syllable listening secondary tasks. Nevertheless, previous studies revealed that articulatory suppression and tapping do not differ in the level of disruption they entail in several non-linguistic cognitive tasks such as digit size judgment tasks^34^ and task switching^35,36^, suggesting that those secondary tasks do not drastically vary in the amount of cognitive load they imply. Furthermore, the only difference between the syllable production and tapping tasks in the present study was in the linguistic status of the production (being a syllable or a noise), which also indicates that the cognitive load imposed by those secondary tasks was similar. Finally, performance both in terms of average reaction times and accuracy, and associated variability measures in the comprehension questions did not significantly differ across groups. Taken together, this pattern of results suggests that the level of cognitive load was similar across the three groups^27–30^. Still, we cannot entirely rule out the possibility that a larger cognitive load entailed by the syllable production task was affecting prediction and not comprehension. Future work will be needed to deeply explore cognitive load implied by articulatory suppression during reading and whether it can affect prediction specifically.

Our results cannot speak on the nature of the prediction and the role of production in it. Participants in the two control groups certainly built specific lexical predictions of the upcoming noun and its gender. Whether such prediction involves phonological and/or phonotactic representations (of the noun and/or article) remains to be explored (see^37^ and^24,38^ for evidence pro and against prediction involving phonological representations). Without concluding on the nature of the prediction (i.e., the “what”; see^1^), we can assert that the way lexical predictions are built rests on subvocal rehearsal of the verbal input^17^ during context reading, or at least on production processes made inoperative by rehearsal. Interestingly, we can state that the availability of the production system plays a crucial role during context reading (and not only at a late point in time close to the predictable input), given that the articulatory suppression in the SP group took place during context reading and stopped 3 words before the display of the critical word of the sentence. Thus, the role of the production system seems to be predominant when constraining semantic information is gathered from the sentence context. The availability of the production system late in time (a few words before the predictable input) is not sufficient to build up lexical predictions, revealing an important role of production in assembling semantic information from the context. The necessary role of the production system in selecting the most expected word itself cannot be defined given that production was not blocked anymore during such preparatory processes likely to happen a few words before the critical noun-phrase.

The present results are also relevant in regard to the current interest on variability in predictive processes^39,40^. Despite the fact that many researchers agree that readers actively predict upcoming information during sentence comprehension, it is also largely admitted that such predictive processes are prone to variability in participants, task requirement and context^13,15,39^. Many authors agree that predictive processes should be affected by cognitive resources availability and cognitive control^39,41^ but evidence of it is scarce. Ito and colleagues^42^ recently showed that predictive processing was delayed when participants had to perform a secondary working memory task during sentence listening (see also^43^ and^44^ for evidence pro and against an involvement of working memory capacities in prediction). Thus, prediction might not be robust enough to be unaffected by verbal working memory load. Quite the opposite, the present results tend to show some sort of impermeability of predictive processes to cognitive resource availability, given that the expectation effect was largely significant in both the TT and SL groups, despite the concurrent non-verbal secondary task. Note also that the magnitude of the N400 effect elicited by the article in the TT and SL groups is similar to the one reported in a previous experiment using similar paradigm and materials but without double-tasking^20^. Thus, we provide the first piece of evidence that prediction is a cognitive process strong/relevant enough to survive non-verbal double-tasking. This is not to say that comprehenders always predict, but that prediction might be automatic as far as the language production system and verbal working memory are available. Since it could be that our control tasks were not cognitively demanding enough to significantly affect any other process, further research is needed to conclude on the relative automaticity of prediction depending on the type and amount of cognitive load at play. Interestingly, it has been shown that prediction effects are reduced in second language (L2) readers^23,45^, in low literate adults^46^, in children with poor vocabulary^11^ and in older adults^10^. Such reduced prediction effects can arguably be linked to reduced cognitive resource availability (e.g., reduced verbal working memory), but the present results offer an interesting new perspective: The lack of prediction in certain populations might reflect a lack of fast and efficient engagement of production processes during comprehension. This assumption should be tested for a full understanding of the production/comprehension functional link.

Finally, the results observed on the N400 component elicited by the noun are also informative. This N400 component reflects semantic processing of the critical noun, which can be influenced by sentence context through passive resonance, message-level build up, but also through after-effects of prediction (see^23,47^ for extensive discussion). Here, we observed a significant expectation effect on the critical noun in the three groups. This shows that integration of the critical noun was not hindered by the lack of prediction in the SP group. As previously shown in L2 readers, the most expected critical noun was easier to integrate, based on previous sentence context, despite the absence of significant evidence of its active prediction^23,47^. This result is also in line with previous work showing that articulatory suppression does not prevent proper sentence comprehension^48^, and with neuropsychological evidence showing that aphasic patients with highly impaired production skills can have language comprehension somehow preserved (for a review see^49^). Thus, production is necessary for prediction but prediction is not mandatory for proper integration. We can also assert that the unavailability of the production system during sentence context reading is not detrimental for proper integration of the predictable noun. Whether such unavailability would still have negligible effects on word integration if it was to happen during (and not only before) critical word display still has to be explored, given that, in the present study, the production system was not taxed anymore during predictable noun integration.

To conclude, the present study provides the first strong and direct evidence in favor of the hypothesis that prediction is production, showing that the availability of the speech production system during sentence context display is necessary to build up lexical prediction during reading. The major role of the production system for prediction in comprehension can explain the recruitment of production processes during language comprehension.
